# Utility of hand-held ultrasound for image acquisition and interpretation by trained Kenyan providers

**DOI:** 10.1186/s13089-023-00308-7

**Published:** 2023-03-08

**Authors:** Grace Wanjiku, Lindsay Dreizler, Shirley Wu, Janette Baird, Benjamin Wachira

**Affiliations:** 1 Department of Emergency Medicine, The Warren Alpert Medical School of Brown University, 55 Claverick Street Suite 100, Providence, RI 02903 USA; 2 The Warren Alpert Medical School of Brown University, 222 Richmond Street, Providence, RI 02903 USA; 3 Accident and Emergency Department, The Aga Khan University, 3Rd Parklands Avenue, Nairobi, Kenya; 4 Section of Global Emergency Medicine, The Warren Alpert Medical School of Brown University, 55 Claverick Street Suite 101, Providence, RI 02903 USA

## Abstract

**Background:**

Point-of-care ultrasound (POCUS) plays a prominent role in the timely recognition and management of multiple medical, surgical, and obstetric conditions. A POCUS training program for primary healthcare providers in rural Kenya was developed in 2013. A significant challenge to this program is the acquisition of reasonably priced ultrasound machines with adequate image quality and the ability to transmit images for remote review. The goal of this study is to compare the utility of a smartphone-connected, hand-held ultrasound with a traditional ultrasound device for image acquisition and interpretation by trained healthcare providers in Kenya.

**Methods:**

This study took place during a routine re-training and testing session for healthcare providers who had already received POCUS training. The testing session involved a locally validated Observed Structured Clinical Exam (OSCE) that assessed trainees’ skills in performing the Extended Focused Assessment with Sonography for Trauma (E-FAST) and focused obstetric exams. Each trainee performed the OSCE twice, once using a smartphone-connected hand-held ultrasound and once using their notebook ultrasound model.

**Results:**

Five trainees obtained a total of 120 images and were scored on image quality and interpretation. Overall E-FAST imaging quality scores were significantly higher for the notebook ultrasound compared to the hand-held ultrasound but there was no significant difference in image interpretation. Overall focused obstetric image quality and image interpretation scores were the same for both ultrasound systems. When separated into individual E-FAST and focused obstetric views, there were no statistically significant differences in the image quality or image interpretation scores between the two ultrasound systems. Images obtained using the hand-held ultrasound were uploaded to the associated cloud storage using a local 3G-cell phone network. Upload times were 2–3 min.

**Conclusion:**

Among POCUS trainees in rural Kenya, the hand-held ultrasound was found to be non-inferior to the traditional notebook ultrasound for focused obstetric image quality, focused obstetric image interpretation, and E-FAST image interpretation. However, hand-held ultrasound use was found to be inferior for E-FAST image quality. These differences were not observed when evaluating each E-FAST and focused obstetric views separately. The hand-held ultrasound allowed for rapid image transmission for remote review.

**Supplementary Information:**

The online version contains supplementary material available at 10.1186/s13089-023-00308-7.

## Background

The use of point-of-care ultrasound (POCUS) benefits patient screening, the accuracy of diagnosis, and management for a wide range of indications in low- and middle-income countries (LMICs) [1, 2]. Several studies examining patient outcomes in low-resource settings revealed ultrasound findings made significant contributions to treatment plans in medical, surgical, and obstetric care specialties [3–6]. Evidence of the advantages of POCUS as well as improvements in cost and ease of use have contributed to growing interest in applications for ultrasound services in LMICs. However, challenges in training and poor access to ultrasound machines persist around the world. In a survey of healthcare professionals in LMICs in 2015, providers identified lack of training, insufficient access to equipment, and adequate maintenance as the most significant barriers to ultrasound use [6]. In addition, a survey in 2020 of 342 participants at six North American institutions identified lack of training, lack of hand-held devices, and lack of direct supervision as the most important barriers to learning and applying POCUS in practice [7].

**Fig. 1:**
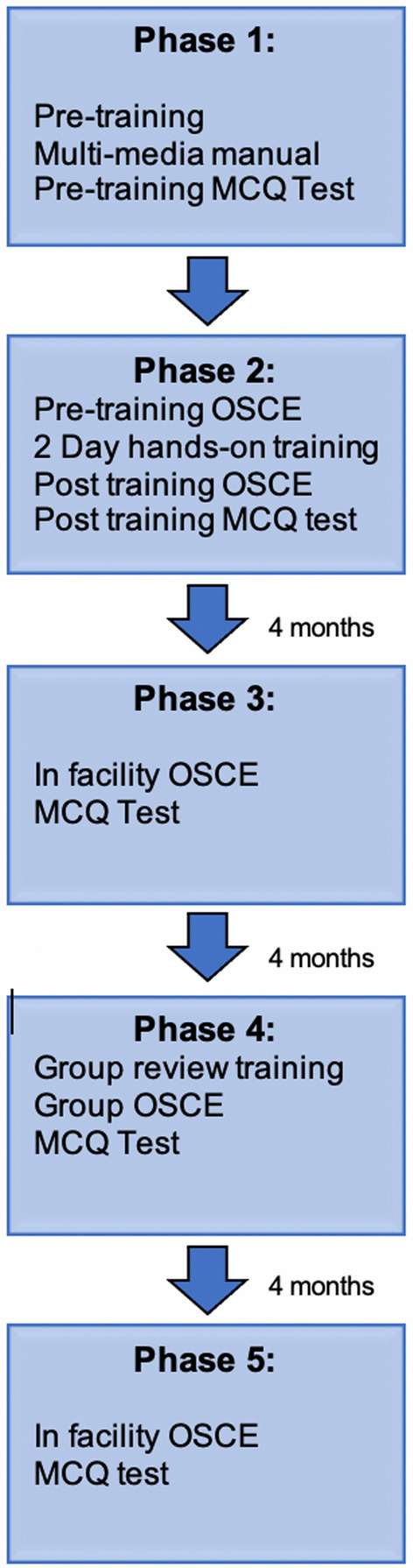
Kenya rural POCUS training program

In 2013, we developed a program to train rural healthcare providers in Kenya on POCUS applications including the Extended Focused Assessment with Sonography for Trauma (E-FAST) and focused obstetric ultrasonography [8]. The program is coupled with ultrasound machine donations and incorporates in-facility evaluations and quality control every four months (Fig. 1). Program evaluation revealed that trainees performed focused obstetrics more than the E-FAST and that less contact with trainers (for re-training and feedback) was correlated with low frequency and quality of scanning [9].

Due to geographic isolation, trainees lack frequent real-time guidance and feedback while scanning at their facilities and have limited interactions with trainers outside of the scheduled sessions 3 times a year. Workforce limitations mean that many trainees are unable to arrange time away from their healthcare facilities to attend training in the capital city. The current ultrasound system that was donated for their use does not allow for image transmission for remote review and quality assurance. Poor infrastructure and limited Internet connectivity further prevent the creation of robust frameworks for image review and feedback [10].

The cost of ultrasound machines has historically prohibited the expansion of POCUS use in LMICs. However, this challenge is more surmountable with the development of more cost-effective hand-held ultrasound models. These ultrasounds present a significantly more affordable option for full-body imaging in low-resource areas. They also improve portability for bedside use. Importantly, these ultrasound models incorporate cloud-based storage systems where images can be immediately uploaded for expert review and feedback.

Several studies have evaluated the efficacy of hand-held ultrasounds and acknowledged their potential benefits [11–14]. One study examined the performance of the Butterfly iQ ultrasound probe in an emergency department in East Africa [13]. While the study identified significant benefits such as cheaper cost and increased portability, they acknowledge further research is needed to assess potential limitations in image quality and machine durability.

The primary aim of this study was to introduce a hand-held ultrasound model into our training program in rural Kenya and to compare image quality and image interpretation by POCUS trainees using the hand-held device compared to the notebook ultrasound model in current use. We also sought to determine the median time required to transmit ultrasound images obtained using the smartphone-connected hand-held ultrasound model through local cell phone networks.

## Methods

This was a pilot study designed as a non-inferiority test of the hand-held ultrasound compared to a notebook ultrasound model currently in use by the Kenyan POCUS trainees. We recruited healthcare providers who had received prior POCUS training and were presenting for follow-up refresher training and testing. All participants were above 18 years of age and provided consent to participate. The study took place in April 2018.

The refresher session entails a full hands-on review of all the point-of-care ultrasound modalities that the trainees have been exposed to before. I.e., The E-FAST and focused first- and second/third-trimester obstetric ultrasound. These exams are carried out on healthy, pre-scanned volunteers who provide informed consent. At the end of the refresher program, trainees are evaluated on their ultrasound scanning skills using a Standardized Observed Structured Clinical Exam (OSCE) (Additional file 1: Appendix A). Specifically, the OSCE assesses the trainee’s clinical skills in detecting free fluid in the pleural, peritoneal, and pericardial cavities as well as ruling out a pneumothorax. Trainees are also assessed on their focused first- and second/third-trimester obstetric scanning skills. These include first-trimester dating using crown-rump length, detecting and measuring the fetal heart rate, identifying the presenting part, locating the placenta, and the measurement of the head circumference and biparietal diameter. Any deficits noted during the OSCE are usually addressed as part of re-training procedures.

During this session, trainees had a 2-h hands-on introduction to a hand-held ultrasound (Butterfly iQ, 2018 Butterfly Network, Inc.) that was connected to an iPhone 8. The trainees practiced using this ultrasound model to perform guided E-FAST and focused obstetric exams. Afterward, we performed the OSCE evaluation twice for each trainee: once using the current notebook ultrasound model (WED, WELLD) and once using the Butterfly iQ probe. The OSCE testing was carried out on a separate group of pre-scanned volunteers. The order of ultrasound use and ultrasound modality testing was randomized to minimize bias from the previous examination. This was achieved by creating a randomized order in which the trainees rotated through different testing stations. Each testing station had a different volunteer/model, half the stations were equipped with a hand-held ultrasound, and the other half were equipped with a notebook ultrasound.

Images were scored independently by three investigators who are credentialed in POCUS according to the American College of Emergency Physician guidelines. Two of these investigators had also completed fellowships in acute and critical care ultrasound. A standardized scoring system was used to grade image quality on a scale of 0 to 4 (0: no meaningful images; 1: poor, not sufficient for interpretation; 2: good, acceptable for interpretation; 3: excellent, minor suggestions for improvement; and 4: outstanding, no suggestions for improvement). This scoring system is currently in use in the Kenya rural ultrasound training program [9] as well as other similar training programs in the African region [14, 15] (Additional file 1: Appendix A).

Image interpretation was scored using a standardized scoring system from 0 to 3: (0: did not acquire images sufficient for interpretation to answer a point-of-care question; 1: correctly acquired images but incorrectly interpreted some of them; 2: correctly acquired images and able to interpret SOME but not all relevant point-of-care questions; and 3: correctly acquired and interpreted images to answer ALL relevant point-of-care questions) (Additional file 1: Appendix A).

Images obtained with the hand-held ultrasound were uploaded to a HIPAA compliant cloud account using a local 3G cell phone network. The median time required for image transmission was documented. Images obtained using the notebook model were saved on the ultrasound machine for review.

### Data analysis

Descriptive analyses on image quality and image interpretation scores were conducted with median and interquartile range (IQR) reported. Wilcoxon tests were conducted to determine differences between the notebook and hand-held imaging systems on overall E-FAST and focused obstetric image quality and image interpretation. A series of Wilcoxon tests were also performed to determine differences between the two ultrasound systems on each separate E-FAST and focused obstetric view, e.g., identifying differences in image quality and image interpretation for Right Upper Quadrant (RUQ) and Left Upper Quadrant (LUQ) views separately. Within each view category (E-FAST and obstetric), the scores for the two internal reviewers were averaged. The score of the external reviewer was used as a validity check for the internal consistency of the two primary internal reviewers. Agreement rates between the two internal reviewers and the external reviewer were calculated for image quality. Agreement within 1 point between the internal and external reviewers was considered as agreement.

### Ethics

The study was approved by the Aga Khan University Research Ethics Committee (REC) and the National Commission for Science, Technology, and Innovation (NACOSTI) Kenya.

## Results

Data were collected from five trainees who conducted a series of ultrasound imaging using the hand-held and notebook ultrasound. Each trainee obtained six E-FAST views and six focused obstetric views on each ultrasound machine, a total of 24 views for each trainee, resulting in 120 total ultrasound images assessed in this study. Image quality was scored on a 0–4 point scale and image interpretation was scored on a 0–3 point scale by three trained raters. Where a trainee obtained multiple images or clips in each view (e.g., RUQ, LUQ), the highest score per view was documented.

For E-FAST views, the overall median image quality score was 3.5 (IQR 3,4) for the notebook ultrasound and 3 (IQR 3,3) for the hand-held ultrasound [*p* = 0.04]. The overall median interpretation scores were 3 (IQR 3,3) for the notebook ultrasound and 3 (IQR 3,3) for the hand-held ultrasound [*p* = 0.67]. For obstetric views, overall median image quality scores were 3.5 (IQR 3,3) for the notebook ultrasound and 3 (IQR 2,4) for the hand-held ultrasound [*p* = 0.18]. The overall median interpretation scores were 3 (IQR 3,3) for the notebook ultrasound and 3 (IQR: 2,3) for the hand-held ultrasound [*p* = 0.06].

Agreement between the internal and external raters reached 53% for E-FAST image quality for the notebook ultrasound and 57% for the hand-held ultrasound. For obstetric views, agreement on image quality was 80% for the notebook and 83% for the hand-held ultrasound.

A series of Wilcoxon tests were conducted on differences in image quality and image interpretation by ultrasound system for the specific E-FAST and obstetric views as shown in Table 1. There was no significant difference between the two ultrasound systems on median image quality and interpretation scores across the separate E-FAST and obstetric ultrasound views.

**Table 1:** Image quality and interpretation scores by ultrasound view and ultrasound type

Ultrasound view	Image quality median (IQR)		*P* value	Image interpretation median (IQR)		*P* value
	Butterfly iQ	WELLD		Butterfly iQ	WELLD	
EFAST						
RUQ	3 (3,3)	4 (4,4)	0.14	3 (3,3)	3 (3,3)	1.00
LUQ	3 (2,4)	3 (3,4)	0.29	3 (3,3)	3 (3,3)	0.52
Suprapubic	3 (2,3)	4 (2,4)	0.44	3 (3,3)	3 (3,3)	0.91
Pneumothorax	3 (3,3)	3 (3,4)	0.61	3 (3,3)	3 (3,3)	0.45
PSL	3 (2,3)	3 (3,3)	0.57	3 (3,3)	3 (3,3)	0.56
Subxiphoid	3 (2,3)	4 (4,2)	0.40	3 (3,3)	3 (3,3)	0.57
Obstetrics						
Sagittal uterus	3 (3,4)	4 (4,4)	0.19	3 (2,3)	3 (3,3)	0.21
Fetal presentation	4 (3,4)	3 (2,4)	0.38	3 (3,3)	3 (3,3)	0.44
Placenta location	3 (3,4)	3 (3,4)	1.00	3 (3,3)	3 (3,3)	0.44
FHR	3 (3,3)	3 (3,3)	0.71	3 (3,3)	3 (3,3)	1.00
BPD	3 (2,3)	3 (2,4)	0.83	3 (2,3)	3 (3,3)	0.53
HC	3 (2,3)	3 (2,4)	0.83	3 (2,3)	3 (3,3)	0.61

*IQR* interquartile range, *RUQ* right upper quadrant, *LUQ* left upper quadrant, *PSL* parasternal long axis, *FHR* fetal heart rate, *BPD* biparietal diameter, *HC* head circumference

Images and short clips obtained using the smartphone-connected hand-held ultrasound were uploaded to the associated cloud storage using a local 3G-cell phone network. Upload times were 2–3 min.

## Discussion

After a 2-h introduction to Kenyan rural POCUS trainees, we found the hand-held ultrasound to be non-inferior to their notebook ultrasound on overall measures of E-FAST image interpretation, focused obstetric image quality, and interpretation. However, the hand-held ultrasound was found to be inferior in overall measures of E-FAST image quality. When comparing separate imaging views, no statistically significant difference was found between the notebook ultrasound compared to the hand-held ultrasound.

Our study findings are comparable with results from a single-center prospective observational study with 307 participants in Sierra Leone where a hand-held ultrasound device was found to be as reliable as the traditional ultrasound device in structured obstetric ultrasound exams [14]. The Sierra Leone study compared a hand-held smartphone-based ultrasound using a US-304 convex probe with 64 elements (3–5 MHz; Lequio Power Technology, Naha, Okinawa, Japan) with a conventional full-featured ultrasound with a 3–5-MHz convex probe and a 7–10-MHz trans-vaginal probe (Mindray SD-10, Shenzhen, China).

Despite our findings showing the non-inferiority of the smartphone-connected hand-held ultrasound, trainees brought up several challenges such as difficulty with the iPhone screen size, especially with the need to use a split screen to calculate fetal heart rate. Second, trainees were challenged by the lack of built-in fetal biometric parameters which were not available at the time of this study. It is possible that optimization of the obstetric modality coupled with the ability to use the hand-held probe connected to a larger screen might further improve image quality and interpretation for obstetrics.

Our trainees underperformed on E-FAST image quality scores when using the hand-held ultrasound. The short 2-h introduction to the hand-held ultrasound may have played a part in this. In addition, our previous study in the same population of trainees showed that they do not perform the E-FAST as often as they perform focused obstetrics [9]. However, once they obtained the images, their interpretation of those images was similar when using the hand-held compared to the notebook ultrasound.

There are notable limitations in this study. The sample size of 120 images obtained by 5 trainees was small. The small sample size was further reduced when we separated and evaluated specific ultrasound views, e.g., RUQ and LUQ. Though the study size is small, it has the benefit of comparing images acquired by multiple novice POCUS trainees. Other studies comparing the efficacy of hand-held and traditional ultrasound devices have relied on comparing the images among a single experienced ultrasonographer [11, 12]. The external validity of the study is limited by the fact that we compared the hand-held ultrasound to a particular notebook ultrasound system that is in current use in rural Kenya. It is possible that other notebook or cart-based ultrasound systems might perform differently. In addition, the study was performed on healthy pre-scanned volunteers, the trainees had just undergone a refresher course, and their image interpretation did not have immediate clinical implications. A different study approach involving trainees in a clinical setting making clinical decisions based on their ultrasound results will further shed light on the utility of different ultrasound systems in patient care.

Finally, the percent agreement between the internal and external rater was good for obstetric image quality (80% for the notebook ultrasound and 83% for the hand-held ultrasound) but poor for E-FAST image quality (53% for the notebook ultrasound and 57% for the hand-held ultrasound). This affects our interpretation of the E-FAST image quality results. The agreement in obstetric image quality results is comparable to the study by Kodaira et al. in Sierra Leone [14]. In studies where inter-rater agreement is low, a third expert rater can be used to adjudicate the differences [16].

This study found that it took 2–3 min to upload ultrasound images from the hand-held device to a secure cloud storage platform. Image upload and transmission were achieved via a local 3G cell phone network. Hand-held devices that allow for rapid image transmission through local cell phone networks have great potential to expand POCUS training through remote image review and feedback [17].

## Conclusion

In this non-inferiority trial, the use of a hand-held ultrasound was found to be non-inferior to a traditional notebook model for focused obstetric image quality, obstetric image interpretation, and E-FAST image interpretation, and found to be inferior to the notebook model for E-FAST image quality. When segregated into different ultrasound views, the hand-held ultrasound was found to be non-inferior for each of the views evaluated. In addition, the hand-held device allowed for rapid image transmission via local cell phone networks. This is highly desirable for POCUS training programs in low-resource settings seeking to establish frameworks for remote image review and feedback. Future studies could test the performance of hand-held ultrasounds which now have improved specifications among a larger group of trainees. Further, studies that include clinical implications will provide useful data on the impact of hand-held ultrasound use on patient outcomes. We believe that this will inform current efforts to improve access to POCUS training in low-resource settings.

## Supplementary Information

**Additional file 1.** Appendix A: Observed Structured Clinical Exam Assessment (OSCE) Form.

## Data Availability

The datasets used and analyzed during the current study are available from the corresponding author on reasonable request.

## Abbreviations

POCUS: Point-of-care ultrasound
OSCE: Observed structured clinical exam
E-FAST: Extended focused assessment with sonography for trauma
LMICs: Low- and middle-income countries
IQR: Interquartile range
RUQ: Right upper quadrant
LUQ: Left upper quadrant
PSL: Parasternal long axis
FHR: Fetal heart rate
BPD: Bi-parietal diameter
HC: Head circumference
