# Supplementary material for: Utility of hand-held ultrasound for image acquisition and interpretation by trained Kenyan providers
Source: Ultrasound J. 2023 Mar 8;15:12. doi: 10.1186/s13089-023-00308-7 (PMC9995612; doi:10.1186/s13089-023-00308-7)
Supplement: Supplementary file 1 — Additional file 1. Appendix A: Observed Structured Clinical Exam Assessment (OSCE) Form. [file 13089_2023_308_MOESM1_ESM.docx]

**Observed Structured Clinical Exam Assessment Form**

**Trainee Name: ____________________________________________________________**

**Designation: ______________________________________________________________**

**Date: ____________________________________________________________________**

| Scanning | | PO | OR | AN | IN | IM |
| --- | --- | --- | --- | --- | --- | --- |
| Cardiac-Subxiphoid | |  |  |  |  |  |
| Cardiac-PSL | |  |  |  |  |  |
| FAST-RUQ | |  |  |  |  |  |
| FAST-LUQ | |  |  |  |  |  |
| FAST-Suprapubic | |  |  |  |  |  |
| Thoracic-Pneumothorax | |  |  |  |  |  |
| Ob – Foetal Heart Rate | |  |  |  |  |  |
| Ob - Sagittal Uterus | |  |  |  |  |  |
| Ob - Presentation | |  |  |  |  |  |
| Ob – Placental Location | |  |  |  |  |  |
| Ob – Head Circumference Measurement | |  |  |  |  |  |
| Ob – Biparietal Diameter Measurement | |  |  |  |  |  |
|  |  |  |  |  |  |  |

**Scoring System**

Pre-scan and most of Post-scan, unless otherwise specified

1=Yes, done correctly

0=No, not done correctly

PO: POsition

1=the probe was placed on the correct position on the patient’s body

0=the probe was placed on the incorrect position

OR: ORientation

1=the selection marker was pointing in the correct direction

0=the selection marker was pointing in the incorrect direction

AN: ANatomy

3=correctly identified and named ALL structures pertinent to image

2=correctly identified and named SOME structures pertinent to image, did not know all

1=correctly identified and named some structures but INCORRECTLY identified or named others

0=could not identify or name pertinent structures or named them all incorrectly

IN: INterpretation

3=correctly acquired and interpreted images to answer ALL relevant point-of-care questions

2=correctly acquired images and was able to interpret SOME but not all relevant point-of-care questions

1=correctly acquired images but INCORRECTLY interpreted some of them

0=did not acquire images sufficient for interpretation to answer point-of-care question

IM: IMage quality

4=outstanding images, no suggestions for improvement

3=excellent images, minor suggestions for improvement

2=good images, acceptable for interpretation

1=poor images, some anatomy discernible but not sufficient for interpretation

0=no meaningful image generated

CO=COmmunication

3=Excellent communication, communicated well with the patient/family, explaining and not overstating findings

2=Good communication, communicated some findings with the patient/family

1=Poor communication, explained some findings incorrectly or overstated findings

0=No communication
